# Feasibility of sustained response through long-term dosing in food allergy immunotherapy

**DOI:** 10.1186/s13223-017-0224-7

**Published:** 2017-12-21

**Authors:** Sandra Andorf, Monali Manohar, Tina Dominguez, Whitney Block, Dana Tupa, Rohun A. Kshirsagar, Vanitha Sampath, R. Sharon Chinthrajah, Kari C. Nadeau

**Affiliations:** Sean N. Parker Center for Allergy and Asthma Research at Stanford University, 269 Campus Drive, CCSR 3215, MC 5366, Stanford, CA 94305-5101 USA

**Keywords:** Desensitization, Food allergy, Maintenance, Oral immunotherapy

## Abstract

**Background:**

Clinical trials using oral immunotherapy (OIT) for the treatment of food allergies have shown promising results. We previously demonstrated the feasibility of desensitization for up to 5 food allergens simultaneously through OIT. In this observational study, we report the findings of long-term follow-up (LTFU) of the participants treated through a single site OIT phase 1 trial.

**Methods:**

The participants (n = 46) were followed up to 72 months since the time they reached 2 g maintenance dose per food in the initial phase 1 trial. During the long-term maintenance dosing, participants continued or reduced the initial maintenance dose of food allergen protein to high (median 2 g protein) vs. low (median 300 mg protein). Participant follow-up included clinical monitoring, standardized OFCs, and in some cases, skin prick tests and measurement of allergen-specific IgE and IgG_4_.

**Results:**

Irrespective of the high vs. low long-term maintenance dose during LTFU, all participants were able to ingest 2 g protein of each food allergen protein during OFCs performed at the end of our LTFU.

**Conclusion:**

Our LTFU cohort of food OIT participants from a single site, phase 1 OIT study, supports the feasibility of sustained desensitization through long-term maintenance dosing.

*Trial registration* Registry: Clinicaltrial.gov. Registration numbers: NCT01490177 (original study); NCT03234764 (LTFU study). Date of registration: November 29, 2011 (original study); July 26, 2017 (LTFU study, registered)

**Electronic supplementary material:**

The online version of this article (10.1186/s13223-017-0224-7) contains supplementary material, which is available to authorized users.

## Background

Food allergy affects 8% of children and 5% of adults in the United States [1, 2]. The current standard of care for these food-allergic individuals is complete avoidance of the offending foods and ready access to injectable epinephrine in the event of accidental exposure [3–6]. Considering the increasing incidence of food allergies over the past two decades [2], and the potentially life-threatening anaphylactic response these allergies can manifest on accidental ingestion [7], there is an urgent need for an effective therapy to treat food allergy.

To date, clinical trials using oral immunotherapy (OIT) for the treatment of food allergies have shown promising results [8, 9]. Desensitization to peanut, cow’s milk, egg, and wheat has been shown to be feasible [10]. [Desensitization: a temporary increase in the threshold for allergen reactivity, requiring continued, regular allergen exposure. Continued/sustained desensitization: persistence of desensitized status over long-term maintenance dosing requiring continued allergen exposure.] A phase 1 OIT study previously conducted by our group demonstrated the safety and feasibility of simultaneous desensitization to multiple (up to 5) food allergens, viz. almond, cashew, egg, hazelnut, milk, peanut, pecan, sesame, and walnut [11].

In a recent meta-analysis of food allergy immunotherapy [12] the authors concluded that food allergen immunotherapy may be effective in achieving desensitization. However, currently available data from a very limited number of long-term follow-up (LTFU) studies following participants from a single allergen-specific immunotherapy (oral/sublingual) [13–19] fail to guide providers to come up with clear recommendations on maintenance dosing after successful completion of a given immunotherapeutic protocol toward continued desensitization. Thus additional long-term studies following OIT are of value, and can increase our understanding of dosing regimens that may sustain desensitization for single and multiple allergens, especially in a realistic patient setting outside of well-defined clinical trials.

In this observational study, we followed participants for up to 72 months who were successfully desensitized to single or multiple (up to 5) food allergens through OIT at our site [11]. The objective of our observational study was to evaluate the feasibility of sustained desensitization with reduced (300 mg–2 g) long-term maintenance dosing. The findings from this study demonstrate the feasibility of sustained desensitization on lowering the dose and altering the frequency of consumption.

## Methods

### LTFU study design and participants

This LTFU study received ethical clearance from Stanford IRB, and was open for enrollment to all the participants, who had successfully completed a phase 1 study under the Investigational New Drug (IND) (Trial registration: Clinicaltrial.gov NCT01490177 [11]) in our clinic. The details on the original phase 1 study protocol and results have been published on a subset of peanut-allergic individuals [11]. Furthermore, Quality of Life studies have been published for this study [20] and a similar OIT trial with 16 weeks of adjunct omalizumab [21, 22]. After achieving successful maintenance dosing in the initial phase 1 study (i.e. on reaching the maintenance dose of 2 g for the first food in their OIT in the original phase 1 trial), the participant was enrolled in the LTFU study. At that point, the clinical team performed skin tests, blood tests and reviewed the participant’s past research data. As long as there were no safety issues (i.e. no moderate to severe allergic reactions in the last 6 months), and the skin tests and blood specific IgE tests showed decreases, the clinical team, together with the patient and family, made a team decision to allow the participant to either continue to ingest the “high” maintenance dose (median 2 g protein of each food allergen in their OIT) or decrease to the “low” (median 300 mg of each food allergen in their OIT) maintenance dose after completion of the phase 1 trial. In summary, the long-term maintenance dose was chosen as a team approach between the participant and the clinical team rather than by personal preference. The major potential source of bias in this observational, not a prospective, randomized, controlled study was -low vs high LTFU dose. Reasons for decreasing doses to a low dose of each food allergen included ease of dosing, improved compliance, and dietary preference. No LTFU maintenance dose decrease was made for safety reasons or adverse symptoms. LTFU visits were conducted every 6–12 months up to month 98 after the start of OIT, which corresponds to a maximum of 72 months after reaching the 2 g maintenance dose for the first food in the original trial (median: 48.4 months, interquartile range 11.4 months) until May 2017. After completion of the original phase 1 trial, the participants switched from food flours/powders to food equivalents based on exact protein amounts for each specific food allergen. Some participants also took their maintenance dose every other day instead of every day. Adverse events were documented as per CTCAE v4.03 criteria [23] and in accordance with regulatory guidelines. An independent Data Safety and Monitoring Board (DSMB) met every 6–12 months to review participant safety data. All participants were required to carry reaction medications.

### Oral food challenges, skin prick tests, and serological analysis

Standardized OFCs using validated and published techniques [24] were performed for each of the participant’s food allergens by a board-certified allergy and immunology specialist at each follow-up visit at the Sean N Parker Center for Allergy and Asthma Research at Stanford University, and in some cases, at the respective local allergist’s office. For each allergen, OFCs were done in a staged approach with approximately 150, 300, 600, 1200, and 2000 mg of each food allergen on separate days. Skin prick tests (SPTs) were performed as per the method in the original phase 1 trial [11].

Allergen-specific IgE and IgG_4_ levels were measured at Johns Hopkins Allergy and Clinical Immunology Reference Laboratory by ImmunoCAP fluorescence enzyme immunoassay (Thermo Fisher scientific/Phadia, Waltham, MA).

### Statistical analysis

The analysis was performed using the statistical language R (version 3.2.3). To evaluate the association between SPT wheal diameter and maintenance dose (low vs. high), a mixed effect linear model was fit controlling for months since maintenance was reached with random effects for patient and allergen type. A similar model was fit for the ratio of IgG_4_ to IgE (log10).

Differences between Kaplan–Meier curves, depicting the timeline of participant’s continuing on a high vs. a low maintenance dose were assessed using a log-rank test (*R* package ‘Survival’ [25, 26]).

## Results

### LTFU study participants

A total of 46 out of 48 participants from the original single-site, phase 1 OIT trial enrolled in this LTFU study (thus the enrollment rate = 95.8%). Of these 46 LTFU participants, 1 withdrew early secondary to issues related to inability to travel to the site. The characteristics of the participants at baseline are summarized in Table 1. Participants treated with OIT to 9 different food allergens (almond, cashew, egg, hazelnut, milk, peanut, pecan, sesame, walnut) were included in this study (Table 1). Most of the participants (21, 45.7%) were desensitized to only one food (i.e. peanut); while 6 participants (13%) ingested long-term maintenance doses of 5 different foods. The analysis of combination of foods administered to our multi-OIT participants suggested coexistence of allergies to egg and milk, and walnut and pecan (Additional file 1: Figure S1), which concurs with the previously published findings [27].

**Table 1:** Participant demographics at the start of LTFU study

Characteristics	ITT participants
Number of ITT participants	46
Drop outs	1
Followed up time in months after reaching 2 g maintenance dose in phase 1 trial, median (range)^a^	48 (27–72)
Sex, n (%)	
Male	24 (52%)
Female	22 (48%)
Age in years, median (range)	10.6 (6.4–46.9)
With other atopic conditions (%)	
Atopic dermatitis	54
Atopic rhinitis	56
Asthma	56
Participants with number foods in OIT (n, %)	
1 food	21 (45.7%)
2 foods	7 (15.2%)
3 foods	9 (19.6%)
4 foods	3 (6.5%)
5 foods	6 (13%)
Participants with certain food in OIT, n (%)	
Almond	5 (10.9%)
Cashew	13 (28.3%)
Egg	9 (19.6%)
Hazelnut	3 (6.5%)
Milk	8 (17.4%)
Peanut	38 (82.6%)
Pecan	8 (17.4%)
Sesame	5 (10.9%)
Walnut	15 (32.5%)

^a^The follow-up time does not include the dropout but only the other 45 participants

### Long-term maintenance doses

**Fig. 1:**
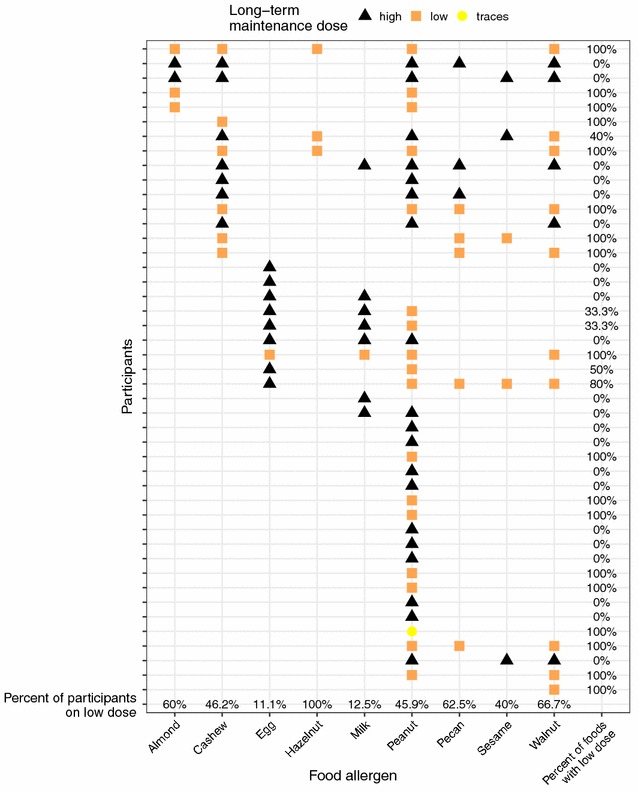
Long-term maintenance doses at the end of LTFU: high vs. low long-term maintenance dose at the end of the follow-up study per participant for each of their respective offending foods administered as a part of OIT is depicted

The long-term maintenance dose information per food per participant at the end of our follow-up study is summarized in Fig. 1.

Of the 25 participants with more than one food in their OIT, 40% (10 participants) were on a low and 40% (10 participants) were on a high long-term maintenance dose for all foods at the end of our follow-up study. Egg (1 of 9 participants on low dose, 11.1%) and milk (1 of 8 participants on low dose, 12.5%) were the foods that were ingested most often at a high dose since these allergens are common ingredients in many foods.

One participant stopped eating peanut after 25 months on a high maintenance dose. That participant however ate foods that contained small amounts (about 50 mg a day) of peanut at the conclusion of our study.

**Fig. 2:**
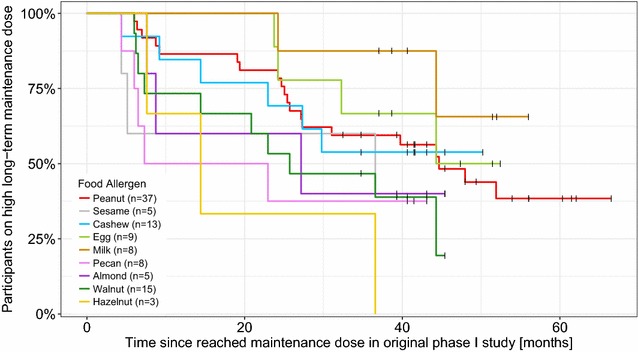
Kaplan–Meier curves of participants on a high maintenance dose over time stratified by allergen: The percentage of participants per allergen continuing a high long-term maintenance dose over time is shown. Black censor lines indicate the time point, where the participants on a high dose were at the conclusion of our follow-up study

The percentage of participants, who continued the high long-term maintenance dose per allergen as opposed to low long-term maintenance dose over time is shown in Fig. 2. Six participants, who decreased their long-term maintenance dose to low, increased it back to high after varying times on the low dose. The time point of only the initial transition from the high into the low maintenance dose is shown in Fig. 2.

### Adverse events during LTFU

Adverse events related to allergic reactions were recorded for the ITT population during the LTFU and are summarized in Tables 2, 3 and Additional file 2: Table S1. In total 1207 reactions were recorded (2.29% of maintenance doses). Of those, 1073 reactions (88.9%) were mild and 129 (10.69%) were classified as moderate. There were 5 severe events (0.41% or all reactions) but these were severe based on nasal congestion and skin symptoms only. There were no fatal or serious adverse events. There were no anaphylactic reactions requiring the use of epinephrine. A key finding was that the frequency of allergic adverse events decreased over time (Tables 2, 3). Safety results did not differ based on low vs. high LTFU maintenance dose.

**Table 2:** Summary of allergic adverse events during LTFU

Reaction numbers and % of total reactions	Total	Month 0–12	Month 13–24	Month 25–36	Month 37–48	Month 49–72
Number of participants followed during time period		46	46	46	41	23
Total ITT reactions	1207	822	261	114	10	0
Gastrointestinal	109 (9.03%)	82 (9.98%)	17 (6.51%)	10 (8.77%)	0	0
Mild	107	80	17	10	0	0
Moderate	2	2	0	0	0	0
Severe	0	0	0	0	0	0
Respiratory/thoracic/mediastinal	75 (6.21%)	70 (8.52%)	3 (1.15%)	2 (1.75%)	0	0
Mild	70	70	0	0	0	0
Moderate	5	0	3	2	0	0
Severe	0	0	0	0	0	0
Skin/subcutaneous	403 (33.39%)	251 (30.54%)	99 (37.93%)	53 (46.49%)	0	0
Mild	286	162	75	49	0	0
Moderate	115	88	23	4	0	0
Severe	2	1	1	0	0	0
Eye	25 (2.07%)	16 (1.95%)	7 (2.68%)	2 (1.75%)	0	0
Mild	25	16	7	2	0	0
Moderate	0	0	0	0	0	0
Severe	0	0	0	0	0	0
Cardiovascular	0	0	0	0	0	0
Nasal congestion	286 (23.7%)	166 (20.19%)	85 (32.57%)	35 (30.7%)	0	0
Mild	276	157	84	35	0	0
Moderate	7	6	1	0	0	0
Severe	3	3	0	0	0	0
Other (i.e. anxiety)	309 (25.6%)	237 (28.83%)	50 (19.16%)	12 (10.53%)	10 (100%)	0
Mild	309	237	50	12	10	0
Moderate	0	0	0	0	0	0
Severe	0	0	0	0	0	0

**Table 3:** Summary of allergic adverse events per ITT participant during LTFU

Reaction numbers per ITT participant	Total	Month 0–12	Month 13–24	Month 25–36	Month 37–48	Month 49–72
Number of participants followed during time period	46	46	46	46	41	23
Total reactions per ITT participant median (range)	25 (0–35)	15 (0–21)	5 (0–7)	2 (0–3)	0 (0–1)	0
Gastrointestinal	4 (0–4)	2 (0–3)	0 (0–1)	0 (0–1)	0	0
Mild	2 (0–3)	1 (0–2)	0 (0–1)	0 (0–1)	0	0
Moderate	0 (0–2)	0 (0–1)	0	0	0	0
Severe	0	0	0	0	0	0
Respiratory/thoracic/mediastinal	2 (0–6)	1 (0–4)	0 (0–1)	0 (0-1)	0	0
Mild	1 (0–5)	1 (0–4)	0	0	0	0
Moderate	0 (0–1)	0	0 (0–1)	0 (0–1)	0	0
Severe	0	0	0	0	0	0
Skin/subcutaneous	8 (0–9)	5 (0–47)	2 (0–4)	1 (0–2)	0	0
Mild	7 (0–9)	4 (0–27)	1 (0–2)	1 (0–1)	0	0
Moderate	1 (0–3)	2 (0–20)	1 (0–1)	0 (0–1)	0	0
Severe	0 (0–1)	0 (0–2)	0 (0–1)	0	0	0
Eye	1 (0–2)	0 (0–5)	0 (0–1)	0 (0–1)	0	0
Mild	1 (0-2)	0 (0–5)	0 (0–1)	0 (0–1)	0	0
Moderate	0	0	0	0	0	0
Severe	0	0	0	0	0	0
Cardiovascular	0	0	0	0	0	0
Nasal congestion	6 (0–10)	3 (0–5)	2 (0–4)	1 (0–1)	0	0
Mild	6 (0–10)	3 (0–5)	2 (0–4)	1 (0–1)	0	0
Moderate	0 (0–2)	0 (0–1)	0 (0–1)	0	0	0
Severe	0 (0–1)	0 (0–1)	0	0	0	0
Other (i.e. anxiety)	6 (0–21)	3 (0–44)	1 (0–2)	0 (0–1)	0 (0–1)	0
Mild	6 (0-21)	3 (0–44)	1 (0–2)	0 (0–1)	0 (0–1)	0
Moderate	0	0	0	0	0	0
Severe	0	0	0	0	0	0

### SPTs and serological assessment during LTFU

SPTs and assessment of plasma IgE and IgG_4_ were performed for most participants at various time points over the course of LTFU.

**Fig. 3:**
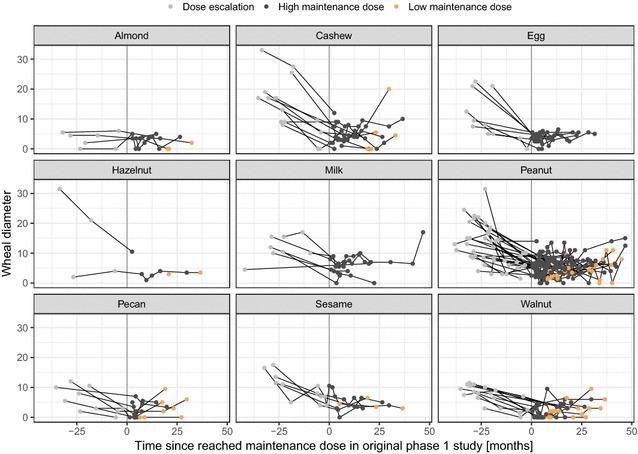
SPT wheal diameters over time: SPT wheal diameters per allergen per participant in the context of time since the maintenance dose of 2 g was reached in the original phase 1 study. Each line represents one participant

The SPT wheal diameter for individual offending foods was significantly decreased during the active phase, and remained low over LTFU (p < 0.05). This phenomenon however was not observed with pecan (p = 0.074), almond (p = 0.71) and sesame (p = 0.13) (Fig. 3).

**Fig. 4:**
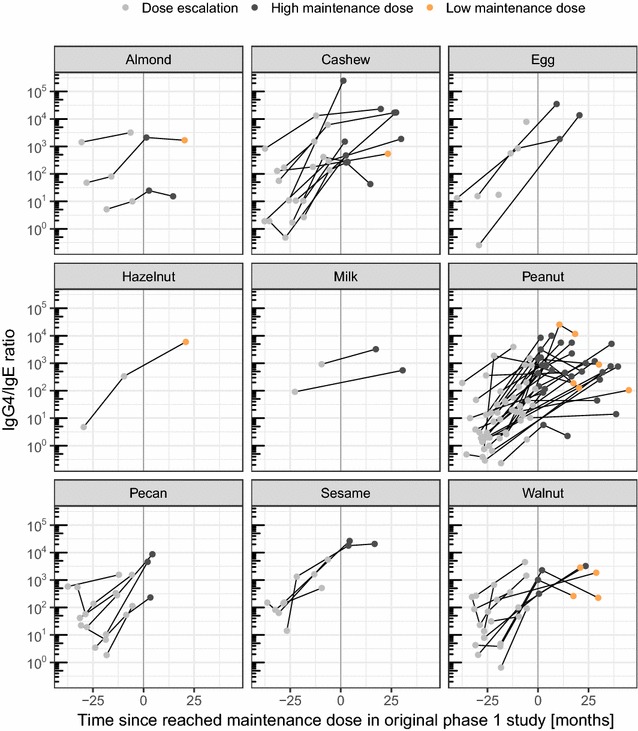
IgG_4_/IgE ratios over time: IgG_4_/IgE ratios per allergen per participant in the context of time since the maintenance dose of 2 g was reached in the original phase 1 study. Each line represents one participant

In concurrence with the previously published findings from the phase 1 study for a subset of peanut-allergic participants [11], a significant trend of increasing allergen-specific IgG_4_/IgE ratios was observed at the end of active phase for all the food allergens. This trend persisted over LTFU (Fig. 4). The statistical significance for the serological readouts could be quantitated only for peanut (n = 20) and cashew (n = 10) (p < 0.01) owing to the limited number of participants with other foods (n < 6) for that the IgE/IgG_4_ was obtained over LTFU.

The SPT wheal diameters (Fig. 3) and IgG_4_/IgE ratios (Fig. 4) per participant and food allergen were analyzed for association with a low vs. a high maintenance dose in a mixed-effects linear model, including the months after reaching the maintenance dose, the food allergen and the participant as covariates. Neither SPT wheal diameter (p = 0.12) nor IgG_4_/IgE ratio (p = 0.15) was significantly associated with a low vs. a high dose in the given dataset.

### Sustained desensitization with either a high or a low long-term maintenance dose

**Fig. 5:**
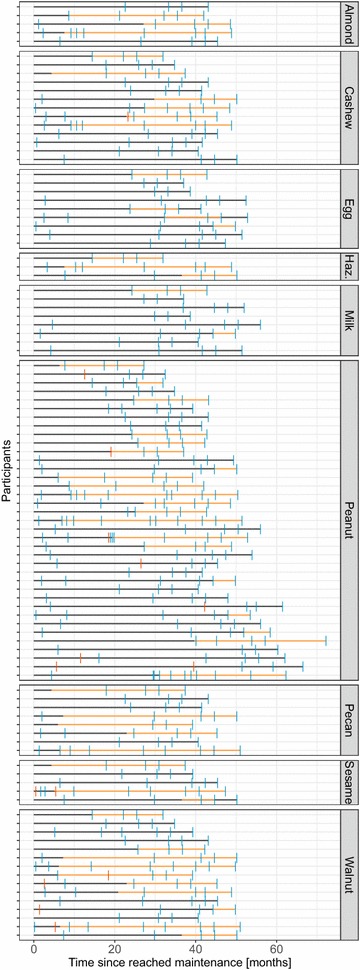
Timeline of high vs. low maintenance doses per participant per allergen: The time of for which each participant was on a high (gray horizontal line) or a low (orange horizontal line) maintenance dose is shown grouped by respective offending foods administered through OIT. The time points of OFCs and the respective outcome are shown as tick marks

The outcome of a standardized, staged OFC performed by trained personnel was used to define the status of desensitization for the LTFU participants. The results of OFCs for each food allergen were analyzed to determine whether a reduced long-term maintenance dose was suitable to sustain desensitization for a given allergen. The time line of the long-term maintenance dose per participant per allergen as well as the time points and outcomes of OFCs are shown in Fig. 5. Each participant on long-term maintenance dosing was able to tolerate 2 g protein or more in a food challenge of their respective food allergens at the end of our follow-up phase, independent of high vs. low long-term maintenance dose. This finding thus demonstrates the feasibility of sustained desensitization even through a reduced long-term maintenance dose.

## Discussion

Immunotherapy for food allergy is an active area of research. Thorough follow-up of study participants is important for determining whether long-term desensitization and maintenance dosing is feasible. Findings from a number of clinical trials exploring the efficacy of oral, sublingual, and epicutaneous administration of various food allergens have been published [8, 9]. However, LTFU studies have been carried out by only a few groups [13–19]. To our knowledge, this study represents one of the longest (up to 72 months past reaching the maintenance dose in OIT) follow-up studies in OIT.

The participants chose to maintain (2 g and over; i.e. ‘high’) or reduce (300 mg to less than 2 g; i.e. ‘low’) their daily maintenance dose of allergens during the LTFU study. The choice to ingest high vs. low dose was not due to safety or allergy symptoms for any participant but rather based upon convenience and taste preference, and was made via a team approach by the participant/caregiver of the participant and clinicians. The main finding of this observational study was that all the participants were able to pass OFCs to 2 g to all their offending foods at the end of our follow-up phase, independent of their low or high long-term maintenance dose. Our results suggest that long-term OIT was possible at a broad age range (specifically, 6.4–46.9 years), and for multiple foods (specifically, almond, cashew, egg, hazelnut, milk, peanut, pecan, sesame, walnut). We also observed that compliance with regular ingestion of food allergens, strong, positive relationships with clinician-parent/participant, and frequent connections between families to support each other favored long-term OIT.

Participant safety is paramount in any allergen immunotherapy study; thus, the participants were counseled frequently on emergency measures. Adverse events were recorded through documented on call services, self-reports, and clinician reports at each visit. Virkud et al. [28] analyzed pooled data from 3 pediatric OIT studies (n = 87) for up to a median of approximately 1.6 years (0.9–3.1 years) and found that 40% of patients had at least one severe event and 12% needed injectable epinephrine. Our data demonstrate a trend in decreased allergic adverse events during the course of the longitudinal study. However, it is important to note that severe allergic reactions (nasal and skin specifically), albeit rare, still occurred randomly during the LTFU, emphasizing the need for constant vigilance and emergency preparedness.

We observed a general trend of decrease in SPT wheal size, and increase in IgE/IgG_4_ ratio in our LTFU cohort.

There were certain limitations to our LTFU study, such as inadequate power to study differences between participants ingesting n = 2 vs. 3 vs. 4 vs. 5 food allergens due to low sample sizes in each group. Also, we did not randomize for low vs. high dosing and left this as a decision for the clinical team with patient-based participation. Future studies using larger, randomized controlled studies are warranted. Despite these limitations, our study underscores the importance of longitudinal, follow-up studies to document whether or not desensitization can be sustained with adequate maintenance dosing over the long term.

## Conclusion

Taken together, these results demonstrate the feasibility of long-term food allergen OIT to up to 72 months after maintenance was reached in OIT (up to 97 months total). The data suggest that food OIT given as long-term maintenance doses is possible and may have long-lasting therapeutic potential with high or low doses.

## Additional files

**Additional file 1: Figure S1.** Coexistence of food allergies in LTFU cohort. The boxes on diagonal indicate the number of participants on maintenance dose of the respective food. The numbers in the rest of the boxes indicate the number of participants with the two foods-one from vertical and the other from horizontal axis in their OIT. The data imply coexistence of certain food allergies.

**Additional file 2: Table S1.** Summary of allergic reactions as adverse events in LTFU study.

## Abbreviations

ITT: intent to treat
LTFU: long-term follow-up
OFC: oral food challenge
OIT: oral immunotherapy
SPT: skin prick test
