# Supplementary figures and images for: Feasibility of sustained response through long-term dosing in food allergy immunotherapy
Source: Allergy Asthma Clin Immunol. 2017 Dec 21;13:52. doi: 10.1186/s13223-017-0224-7 (PMC5738818; doi:10.1186/s13223-017-0224-7)

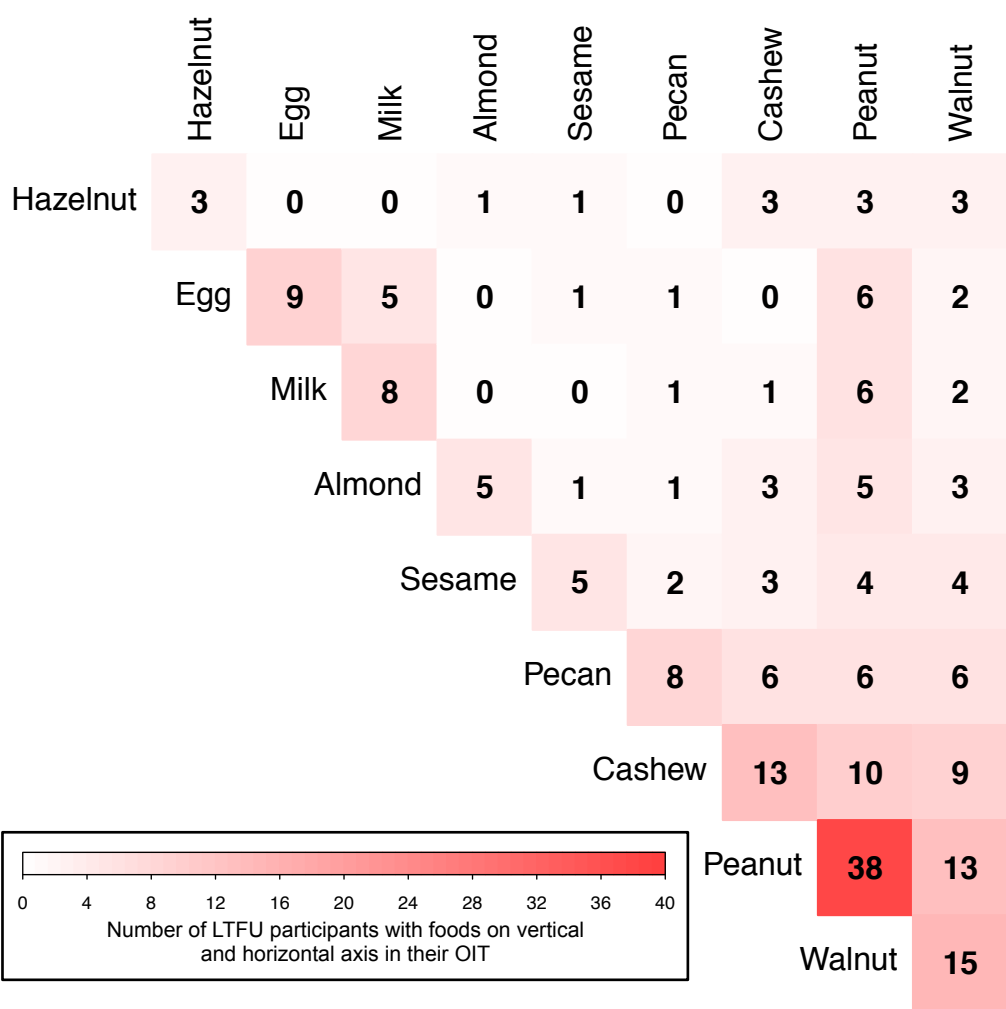

Supplement: Supplementary file 1 — Additional file 1: Figure S1. Coexistence of food allergies in LTFU cohort. The boxes on diagonal indicate the number of participants on maintenance dose of the respective food. The numbers in the rest of the boxes indicate the number of participants with the two foods-one from vertical and the other from horizontal axis in their OIT. The data imply coexistence of certain food allergies. [file 13223_2017_224_MOESM1_ESM.pdf]
