# Supplementary material for: Feasibility of sustained response through long-term dosing in food allergy immunotherapy
Source: Allergy Asthma Clin Immunol. 2017 Dec 21;13:52. doi: 10.1186/s13223-017-0224-7 (PMC5738818; doi:10.1186/s13223-017-0224-7)
Supplement: Supplementary file 2 — Additional file 2: Table S1. Summary of allergic reactions as adverse events in LTFU study. [file 13223_2017_224_MOESM2_ESM.docx]

**Table S1**: Summary of allergic reactions as adverse events in LTFU study

|  | **Number** | **% of maintenance doses** | **% of total reactions** |
| --- | --- | --- | --- |
| Maintenance doses taken | 52,598 |  |  |
| Total reactions | 1,207 | 2.29% |  |
| Mild reactions | 1,073 | 2.04% | 88.9% |
| Moderate reactions | 129 | 0.25% | 10.69% |
| Severe reactions | 5 | 0.01% | 0.41% |
